# Treatment Response, Survival, and Safety of Transarterial Chemoembolization With CalliSpheres^®^ Microspheres Versus Conventional Transarterial Chemoembolization in Hepatocellular Carcinoma: A Meta-Analysis

**DOI:** 10.3389/fonc.2021.576232

**Published:** 2021-03-16

**Authors:** Bin Liang, Joyman Makamure, Shenglei Shu, Lijie Zhang, Tao Sun, Chuansheng Zheng

**Affiliations:** Department of Radiology, Union Hospital, Tongji Medical College, Huazhong University of Science and Technology, Wuhan, China

**Keywords:** hepatocellular carcinoma, CalliSpheres^®^ microspheres, treatment response, survival, safety, meta-analysis

## Abstract

**Background:**

Drug-eluting embolic transarterial chemoembolization (DEE-TACE) is an advance in TACE technique. However, at present there is insufficient evidence to support that DEE-TACE is superior to conventional TACE (cTACE) for hepatocellular carcinoma (HCC). The aim of this meta-analysis is to evaluate the efficacy and safety of TACE with CalliSpheres^®^ microspheres (CSM-TACE) compared with cTACE in patients with HCC.

**Data Sources:**

PubMed, Embase, Web of Science, CNKI and Wanfang Databases were searched to identify relevant articles published before March 26, 2020. The data regarding treatment response, survival profile, adverse events and liver function indexes were retrieved.

**Results:**

A total of 16 studies with 1454 HCC patients (722 treated with CSM-TACE and 732 with cTACE) were included. Patients receiving CSM-TACE had higher 1-month complete response (CR), objective response rate (ORR), disease control rate (DCR) (odds ratio (OR): 2.00, 95% confidence interval (95% CI): 1.29–3.09; OR: 2.87, 95% CI: 2.15–3.83; OR: 2.01, 95% CI: 1.37–2.95, respectively), 3-month CR, ORR, DCR (OR: 4.04, 95%CI: 2.46–6.64; OR: 3.39, 95%CI: 2.45–4.70; OR: 1.71, 95%CI: 1.14–2.55 respectively), and 6-month CR, ORR, DCR (OR: 4.02, 95%CI: 2.26–7.16; OR: 3.00, 95%CI: 2.05–4.38; OR: 2.66, 95%CI: 1.70–4.16 respectively) than those treated with cTACE. Furthermore, CSM-TACE exhibited a trend toward improved progression free survival (hazard ratio (HR): 0.86, 95%CI: 0.67–1.11) and overall survival (HR: 0.79, 95%CI: 0.59–1.07) over cTACE although these differences did not reach statistical significance. In terms of safety, the two TACE treatments showed similar post-treatment pain (OR: 0.84, 95%CI: 0.55–1.28), fever (OR: 0.99, 95%CI: 0.60–1.63), nausea/vomiting (OR: 0.84, 95% CI: 0.60–1.17), as well as 1-month follow-up alanine aminotransferase (Mean difference (MD): −3.66, 95%CI: −10.38–3.07), aspartate aminotransferase (MD: −2.30, 95%CI: −8.91–4.31) and total bilirubin (MD: −0.15, 95%CI: −2.26–1.96).

**Conclusion:**

CSM-TACE displays superior treatment response, non-inferior survival profile and safety over cTACE in HCC patients.

## Introduction

Hepatocellular carcinoma (HCC) is the sixth most common malignancy and the third leading cause of cancer death globally, and more than 50% of HCC cases occur in China (1). Although resection, liver transplantation and ablation are potentially curative options, only a small minority of patients are candidates for these treatments (1, 2). Transarterial chemoembolization (TACE) is the most widely used primary treatment for unresectable HCC and has exhibited encouraging results in terms of survival (1, 2).

Conventional TACE (cTACE) is performed through the intra-arterial injection of single or multiple chemotherapeutic agents with or without ethiodized oil followed by embolization of the tumor-feeding arteries using embolic particles to achieve a strong cytotoxic and ischemic effect on the tumor. Although cTACE elevates the intertumoral concentration of chemotherapeutic agents and decreases the circulating concentration, chemotherapy effect remains limited by limited dosage and uncontrolled release of chemotherapeutic drugs during the procedure (3, 4). Recently, a drug-eluting bead was developed to enhance tumor drug delivery and reduce systemic exposure (5). However, at present there is insufficient evidence that TACE with drug-eluting bead is superior to cTACE for HCC (6, 7).

CalliSpheres^®^ microspheres (CSM), the first microsphere product developed in China, are composed of polyvinyl alcohol hydrogel microspheres with five different sizes (ranging from 100 μm to 1200 μm for different tumor sizes) and some negatively charged functional groups, which are capable of loading a number of positively charged chemotherapeutic drugs (e.g. doxorubicin, pirarubicin, and arsenic trioxide) in a stable fashion by ion-changing in target sites (8–10). Owing to several outstanding features such as satisfactory biocompatibility, adequate physicochemical stability, high drug-loading efficacy and stable releasing profiles (over two weeks), transarterial chemoembolization with CSM (CSM-TACE) is widely applied in treating Chinese HCC patients (8, 11–13). However, comparisons of efficacy and safety between dTACE and cTACE remains controversial (14–16). For instance, Xiang et al. illustrated that CSM-TACE increased objective response rate (ORR), disease control rate (DCR) and progression-free survival (PFS), although it did not reduce adverse events compared with cTACE for HCC (14). On the other hand, Chen at all demonstrated no difference between CSM-TACE and cTACE in short-term response, disease recurrence, complications and side effects (15). To address the dilemma, a meta-analysis comparing CSM-TACE and cTACE in HCC patients is necessary. Therefore, we conducted this meta-analysis including data from 16 articles with 1454 HCC patients to evaluate the treatment response, survival profile and safety between CSM-TACE and cTACE in HCC patients.

## Materials and Methods

### Search Strategy

This meta-analysis was conducted according to the Preferred Reporting Items for Systematic Reviews and Meta-Analysis (PRISMA) guidelines (17). A search of the published literature was performed using the PubMed, Embase, Web of Science, China National Knowledge Infrastructure (CNKI), and Wanfang Databases. The search included articles published up to March 26, 2020, using the following keywords in various combinations: (CalliSpheres^®^ OR CalliSpheres^®^ microspheres OR CSM OR microspheres OR drug-eluting beads OR DEB) AND (liver cancer OR liver cell cancer OR hepatocellular cancer OR hepatic cancer OR hepatoma OR HCC OR liver carcinoma OR liver cell carcinoma OR hepatocellular carcinoma OR hepatic carcinoma). All potentially relevant publications were reviewed, and articles were selected based on predefined selection criteria. References within each study that met the stated selection criteria were manually searched for other potentially relevant studies. Two researchers independently performed a comprehensive systematic search and data extraction, and any discrepancies were resolved by consensus.

### Selection Criteria

The inclusion criteria were as follows: (1) HCC diagnosis was confirmed either histologically or clinically according to the guidelines for diagnosis and treatment of primary liver cancer in China (2); (2) randomized controlled trial, prospective or retrospective observational study; (3) study included two treatment arms: DEB-TACE arm using CalliSpheres^®^ beads and cTACE arm; (4) study contained well-defined outcomes including at least one of the following: (a) treatment response data at 1 month (M1), 3 months (M3), or 6 months (M6), (b) survival data including progression-free survival (PFS) curve, overall survival (OS) curve or hazard ratio (HR) and 95% confidence interval (CI); (5) study was published in English or Chinese. The exclusion criteria were: (1) articles were reviews, letters, case reports, editorials, dissertation, conference paper, as well as expert comments; (2) full text was inaccessible; (3) treatment response data and survival data were missing or unavailable; (4) the article had been withdrawn or citation information was incorrect.

### Data Extraction and Quality Assessment

The data of eligible studies were extracted independently by two investigators, and any disagreement was settled by a third reviewer. The main extracted data included following information: source, first author, published year, sample size in total and each arm, treatment response (complete response (CR), objective response rate (ORR), disease control rate (DCR)) at M1, M3, and M6 post treatment, HR and 95%CI for PFS and OS, PFS, and OS curves, adverse events including pain, fever, nausea and vomiting during post operation, and liver function indexes at M1 post operation, including total bilirubin (TB), alanine aminotransferase (ALT) and aspartate aminotransferase (AST). The methodological quality of eligible studies was evaluated using the Newcastle-Ottawa quality assessment scale (18), based on patient size, selection, study comparability, follow-ups as well as outcomes of studies.

### Statistical Analysis

All statistical meta-analysis was performed using *meta* package in R 3.6.3 software (Comprehensive R Archive Network, USA). The treatment response, survival data and adverse events between CSM-TACE and cTACE were presented as odds ratio (OR) with 95%CI, or hazard ratio (HR) and 95%CI, and the levels of liver function indexes were presented as mean difference (MD) with 95%CI. The pooled analysis of the included studies was carried out using the fixed and random effects model. Heterogeneity between studies was evaluated by I^2^ value. A *P*-value<0.05 or an I^2^>50% was considered as significant heterogeneity. If heterogeneity was not significant, the results of fixed effects model were selected for pooled analysis; if significant heterogeneity existed, the results of random effects model were selected for pooled analysis; Furthermore, sensitivity analysis was performed to explain the possible causes of heterogeneity. The potential publication bias was analyzed by funnel plot and determined by the Egger regression test and Begg & Mazumdar test.

## Results

### Literature Research Process

**Figure 1:**
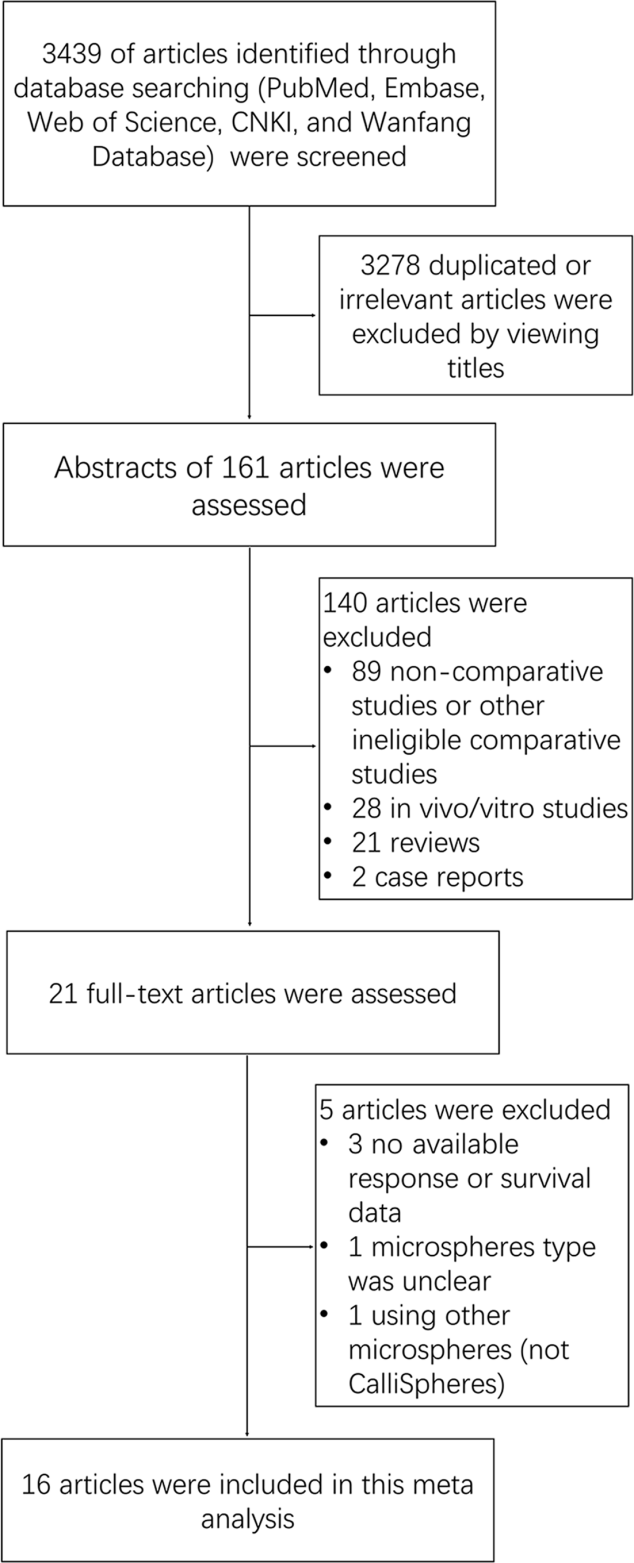
Flow chart.

Our search strategy yielded 3,439 potentially relevant articles, and they were not unique articles (**Figure 1**). After screening by title, reviewing abstract and full-text, 16 articles were eventually included in this meta-analysis. The detailed literature research process was shown in **Figure 1**.

### Characteristics of the Included Studies

The detailed treatment response, PFS, OS, adverse events post operation, and liver function indexes of the 16 studies were summarized in **Tables 1** and **2**. In brief, all studies were published in 2017 or later, and conducted in China; A total of 722 patients were treated with CSM-TACE whereas 732 patients were treated with cTACE.

**Table 1:** Summary of treatment response, PFS, and OS in the studies.

Studies	Treatment arms	No.	Treatment response at M1			Treatment response at M3			Treatment response at M6			PFS	OS
			CR	ORR	DCR	CR	ORR	DCR	CR	ORR	DCR	HR (95%CI)	HR (95%CI)
Liang et al. (19)	CSM-TACE	171	13/107	77/107	96/107	16/82	63/82	68/82	9/54	38/54	48/54	0.882 (0.611–1.274)	0.753 (0.485-1.167)
	cTACE	164	10/124	58/124	108/124	3/55	25/55	46/55	3/45	21/45	36/45		
Duan et al. (20)	CSM-TACE	38	–	–	–	10/38	30/38	30/38	11/38	23/38	27/38	–	–
	cTACE	48	–	–	–	4/48	25/48	33/48	2/48	16/48	16/48		
Ma et al. (16)	CSM-TACE	94	7/57	40/57	52/57	9/44	32/44	34/44	5/27	20/27	24/27	0.820 (0.512–1.312)	0.843 (0.471-1.509)
	cTACE	98	5/82	36/82	73/82	0/40	17/40	34/40	2/31	13/31	25/31		
Zhao et al. (21)	CSM-TACE	42	11/42	34/42	38/42	13/42	32/42	39/42	11/42	32/42	38/42	1.277 (0.659–2.286)	1.010 (0.524-1.947)
	cTACE	47	3/47	26/47	38/47	2/47	21/47	40/47	5/47	26/47	36/47		
Liu et al. (22)	CSM-TACE	14	1/11	8/11	10/11	–	–	–	–	–	–	–	–
	cTACE	9	0/8	1/8	6/8	–	–	–	–	–	–		
Xiao et al. (23)	CSM-TACE	26	–	8/26	18/26	–	–	–	–	–	–	–	–
	cTACE	32	–	4/32	12/32	–	–	–	–	–	–		
Liu et al. (24)	CSM-TACE	31	–	20/31	29/31	–	19/31	27/31	–	–	–	–	–
	cTACE	40	–	18/40	36/40	–	12/40	24/40	–	–	–		
Li et al. (25)	CSM-TACE	34	19/34	30/34	33/34	–	–	–	–	–	–	–	–
	cTACE	33	12/33	25/33	31/33	–	–	–	–	–	–		
Wang et al. (26)	CSM-TACE	45	–	–	–	16/45	36/45	41/45	–	–	–	–	–
	cTACE	45	–	–	–	11/45	34/45	38/45	–	–	–		
Zhou et al. (27)	CSM-TACE	34	–	–	–	–	–	–	8/34	28/34	30/34	–	–
	cTACE	30	–	–	–	–	–	–	3/30	17/30	24/30		
Xu et al. (28)	CSM-TACE	32	–	–	–	4/32	27/32	31/32	–	–	–	–	–
	cTACE	26	–	–	–	2/26	16/26	27/26	–	–	–		
Lin et al. (29)	CSM-TACE	33	9/33	20/33	28/33	–	–	–	–	–	–	–	–
	cTACE	32	7/32	14/32	20/32	–	–	–	–	–	–		
Xiang et al. (14)	CSM-TACE	36	4/25	17/25	24/25	3/15	15/15	15/15	3/9	9/9	9/9	0.326 (0.118–0.899)	0.397 (0.124-1.267)
	cTACE	37	3/28	11/28	25/28	0/8	5/8	6/8	1/7	4/7	5/7		
Wu et al. (30)	CSM-TACE	24	–	–	–	6/24	20/24	22/24	5/24	15/24	20/24	–	–
	cTACE	30	–	–	–	1/30	13/30	20/30	0/30	9/30	17/30		
Ou et al. (31)	CSM-TACE	46	0/46	14/46	31/46	–	–	–	–	–	–	–	–
	cTACE	41	0/41	5/41	22/41	–	–	–	–	–	–		
Chen et al. (15)	CSM-TACE	22	3/17	12/17	16/17	3/17	10/17	13/17	2/17	4/17	10/17	–	–
	cTACE	20	2/16	9/16	13/16	0/16	7/16	12/16	0/16	4/16	10/16		

PFS, progression-free survival; OS, overall survival; CR, complete response; ORR, objective response rate; DCR, disease control rate; HR, hazard ratios; CI, confidence interval; CSM-TACE, CalliSpheres^®^ microspheres transarterial chemoembolization; cTACE, conventional transarterial chemoembolization. “-” indicated the missing data.

**Table 2:** Summary of adverse events post operation and liver function indexes at M1 in the studies.

Studies	Treatment arms	No.	Adverse events post operation			M1 post operation (Mean (SD))		
			Pain	Fever	Nausea/vomiting	ALT (U/L)	AST (U/L)	TB (umol/L)
Liang et al. (19)	CSM-TACE	171	41/171	31/171	17/171	37.1 (26.2)	53.5 (35.5)	17.4 (7.9)
	cTACE	164	29/164	35/164	12/164	31.0 (22.0)	44.7 (29.2)	15.2 (8.3)
Duan et al. (20)	CSM-TACE	38	21/38	21/38	8/38	–	–	–
	cTACE	48	28/48	27/48	22/48	–	–	–
Ma et al. (16)	CSM-TACE	94	34/94	26/94	10/94	37.8 (32.5)	55.4 (39.8)	17.0 (7.6)
	cTACE	98	22/98	14/98	11/98	35.0 (22.2)	47.0 (27.2)	14.5 (9.2)
Zhao et al. (21)	CSM-TACE	42	24/42	16/42	5/42	47.0 (27.8)	62.0 (49.1)	19.0 (11.1)
	cTACE	47	15/47	7/47	2/47	31.0 (19.9)	49.5 (37.1)	18.0 (10.2)
Liu et al. (22)	CSM-TACE	14	0/14	0/14	–	–	–	–
	cTACE	9	2/9	1/9	–	–	–	–
Xiao et al. (23)	CSM-TACE	26	22/26	16/26	3/26	–	–	–
	cTACE	32	28/32	27/32	5/32	–	–	–
Liu et al. (24)	CSM-TACE	31	6/31	10/31	23/31	–	–	–
	cTACE	40	8/40	23/40	37/40	–	–	–
Li et al. (25)	CSM-TACE	34	8/34	–	–	–	–	–
	cTACE	33	9/33	–	–	–	–	–
Wang et al. (26)	CSM-TACE	45	6/45	4/45	13/45	42.6 (10.7)	48.6 (10.8)	–
	cTACE	45	4/45	5/45	12/45	56.6 (10.8)	59.5 (9.1)	–
Zhou et al. (27)	CSM-TACE	34	11/34	–	–	38.8 (19.0)	45.4 (15.3)	16.7 (4.2)
	cTACE	30	20/30	–	–	56.8 (30.8)	60.8 (32.3)	20.9 (8.5)
Xu et al. (28)	CSM-TACE	32	17/32	10/32	8/32	52.3 (4.9)	59.8 (6.2)	24.6 (2.0)
	cTACE	26	21/26	12/26	8/26	57.7 (4.9)	63.3 (6.3)	26.8 (2.2)
Lin et al. (29)	CSM-TACE	33	5/33	3/33	7/33	–	–	–
	cTACE	32	8/32	4/32	10/32	–	–	–
Xiang et al. (14)	CSM-TACE	36	8/36	5/36	2/36	41.2 (24.5)	50.4 (25.3)	17.0 (5.7)
	cTACE	37	6/37	3/37	2/37	38.1 (21.1)	45.3 (24.2)	15.1 (9.6)
Wu et al. (30)	CSM-TACE	24	15/24	–	–	40.5 (22.2)	45.7 (15.5)	16.9 (4.2)
	cTACE	30	26/30	–	–	56.8 (30.8)	60.8 (32.3)	20.9 (8.5)
Ou et al. (31)	CSM-TACE	46	–	–	–	–	–	–
	cTACE	41	–	–	–	–	–	–
Chen et al. (15)	CSM-TACE	22	8/22	8/22	6/22	–	–	–
	cTACE	20	11/20	9/20	9/20	–	–	–

SD, standard deviation; ALT, alanine aminotransferase; AST, aspartate aminotransferase; TB, total bilirubin; CSM-TACE, CalliSpheres^®^ microspheres transarterial chemoembolization; cTACE, conventional transarterial chemoembolization. “-” indicated the missing data.

### Treatment Responses

**Figure 2:**
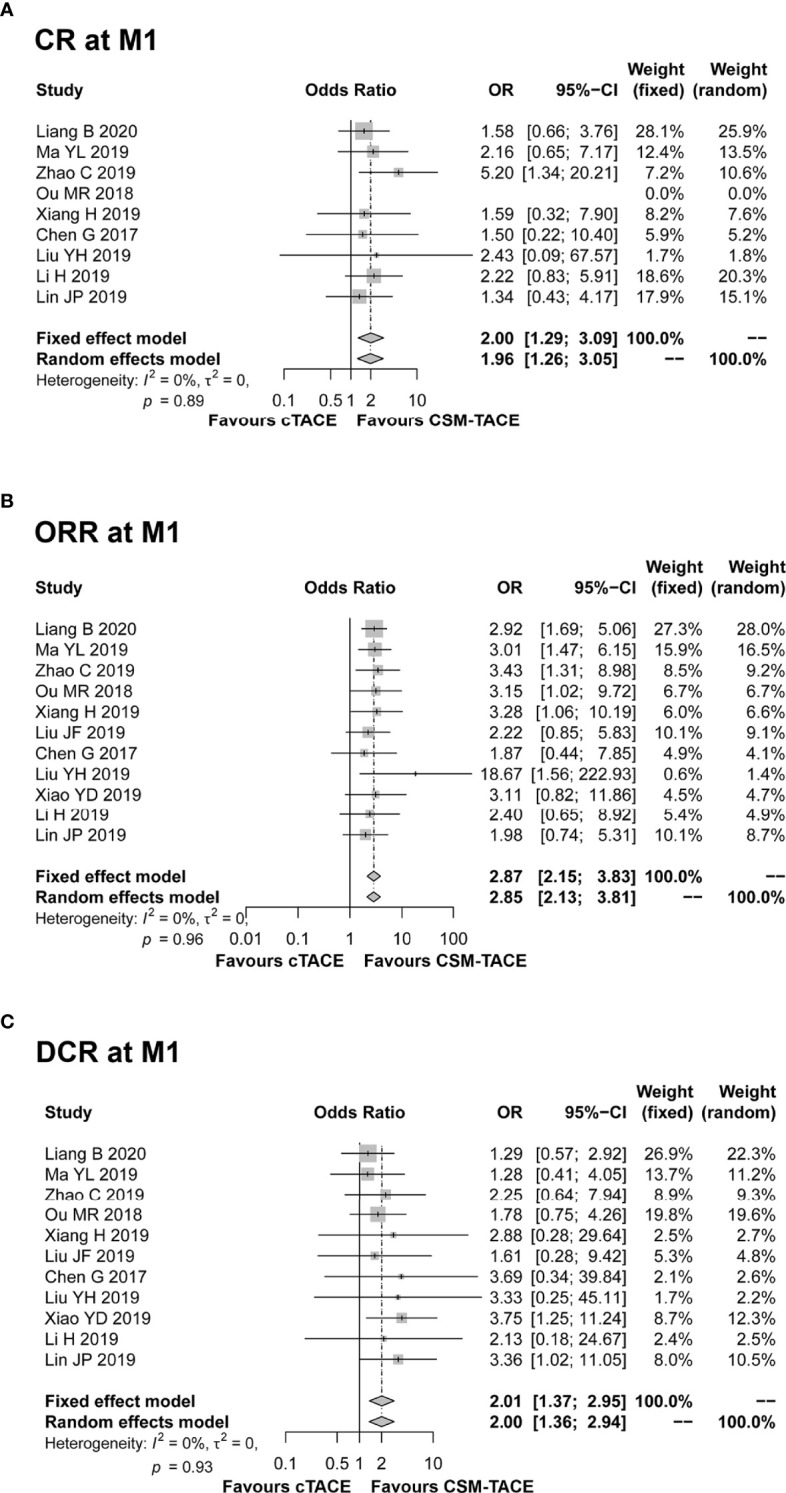
Forest plot comparing CR at M1 **(A)**, ORR at M1 **(B)**, and DCR at M1 **(C)** between CSM-TACE treatment and cTACE treatment. CR, complete response; M1, 1 month; ORR, objective response rate; DCR, disease control rate; CI, confidence interval; cTACE, conventional transarterial chemoembolization; CSM-TACE, CalliSpheres^®^ microspheres transarterial chemoembolization.

**Figure 3:**
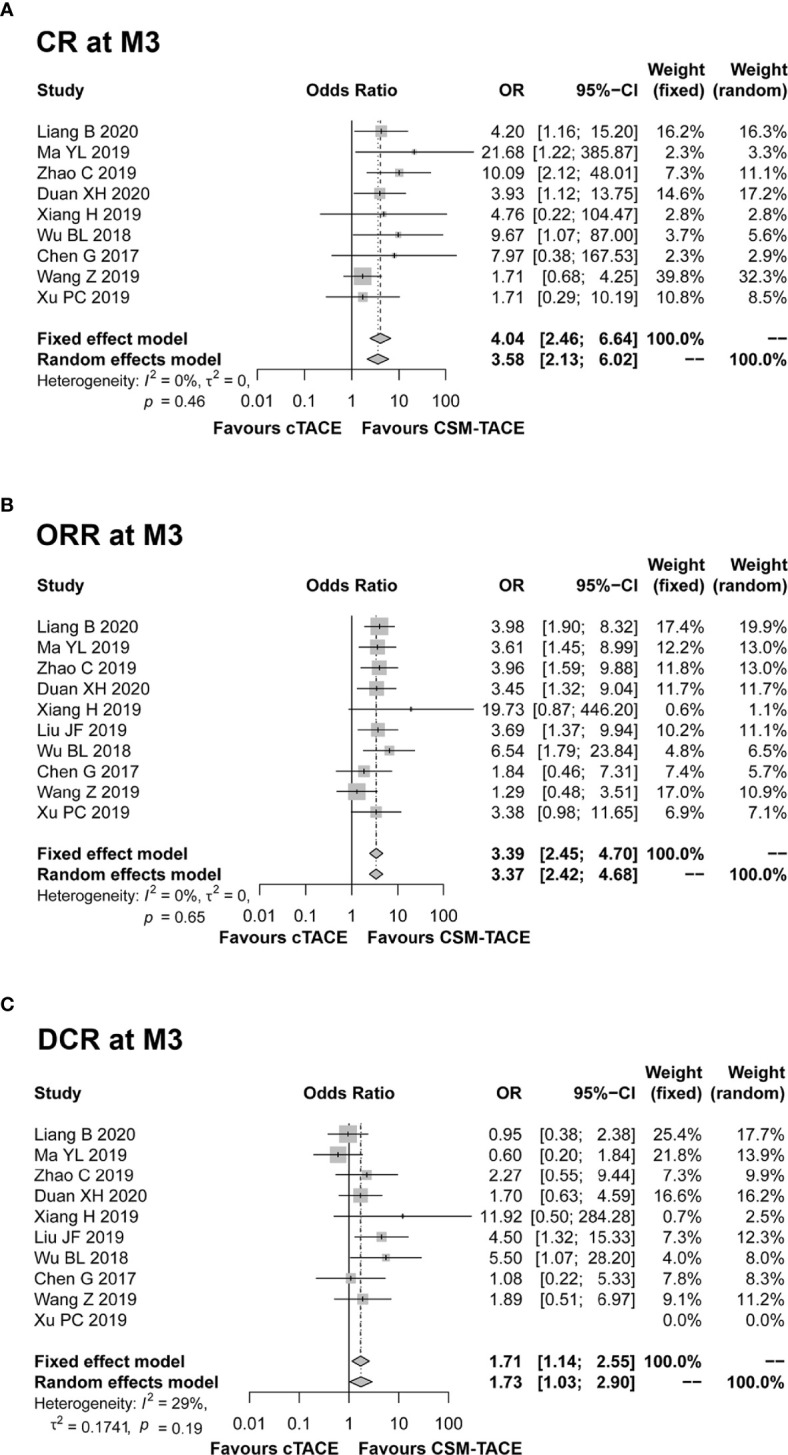
Forest plot comparing CR at M3 **(A)**, ORR at M3 **(B)**, and DCR at M3 **(C)** between CSM-TACE treatment and cTACE treatment. CR, complete response; M3, 3 months; ORR, objective response rate; DCR, disease control rate; CI, confidence interval; cTACE, conventional transarterial chemoembolization; CSM-TACE, CalliSpheres^®^ microspheres transarterial chemoembolization.

**Figure 4:**
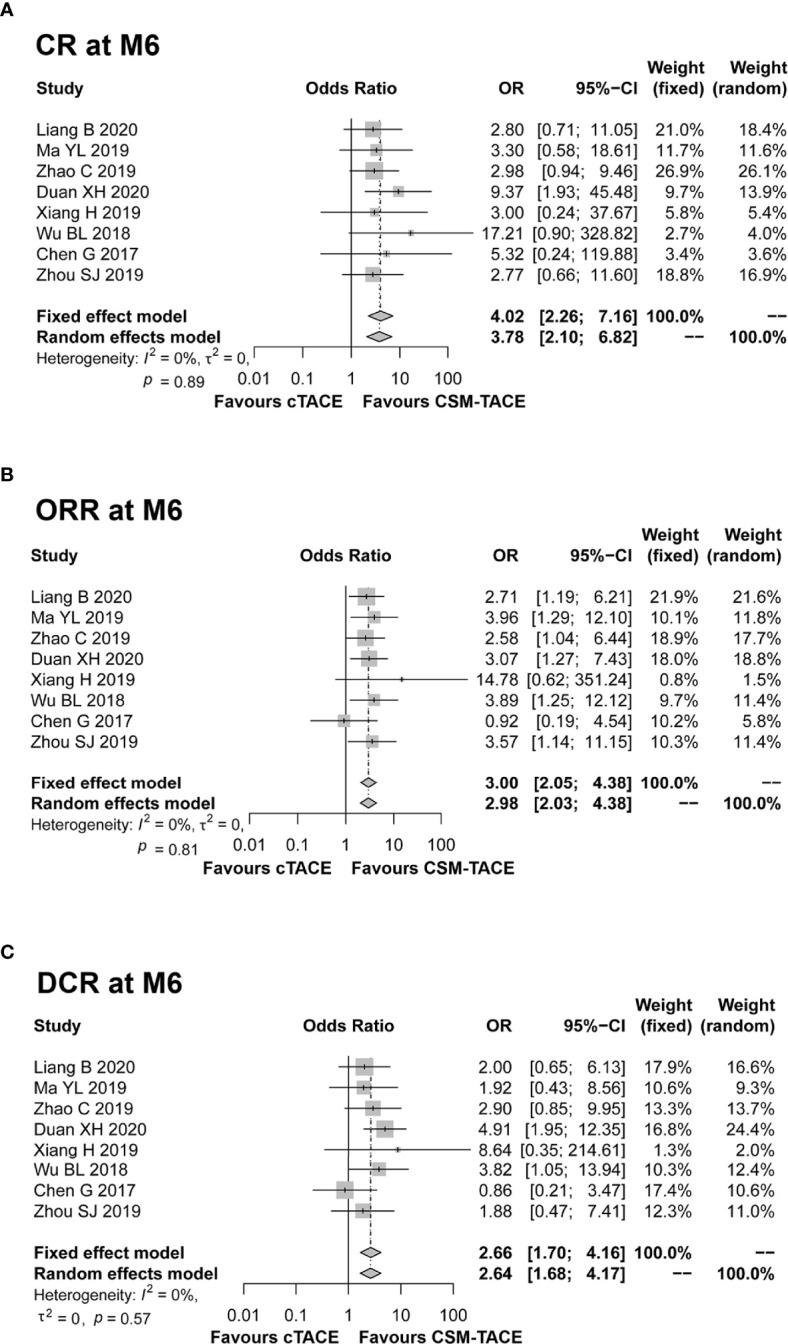
Forest plot comparing CR at M6 **(A)**, ORR at M6 **(B)**, and DCR at M6 **(C)** between CSM-TACE treatment and cTACE treatment. CR, complete response; M6, 6 months; ORR, objective response rate; DCR, disease control rate; CI, confidence interval; cTACE, conventional transarterial chemoembolization; CSM-TACE, CalliSpheres^®^ microspheres transarterial chemoembolization.

A total of 9 studies presented data on CR at M1, and 11 studies provided data on ORR and DCR at M1. The pooled data from these studies showed that CR (OR: 2.00, 95% CI: 1.29–3.09) (**Figure 2A**), ORR (OR: 2.87, 95% CI: 2.15–3.83) (**Figure 2B**) and DCR (OR: 2.01, 95% CI: 1.37–2.95) (**Figure 2C**) at M1 were higher by CSM-TACE treatment than cTACE treatment without significant heterogeneity among studies (all I^2^ = 0%, *P* > 0.05). As for CR at M3, 9 studies reported relevant data, and 10 studies reported data on ORR and DCR at M3. The pooled analysis showed that CR (OR: 4.04, 95% CI: 2.46–6.64) (**Figure 3A**), ORR (OR: 3.39, 95% CI: 2.45–4.70) (**Figure 3B**) and DCR (OR: 1.71, 95% CI: 1.14–2.55) (**Figure 3C**) at M3 were increased by CSM-TACE treatment compared with cTACE treatment without significant heterogeneity among studies (all I^2^ < 50%, all *P >*0.05). With regard to CR, ORR and DCR at M6, eight studies reported relevant data, then the pooled analysis revealed that CR (OR: 4.02, 95% CI: 2.26–7.16) (**Figure 4A**), ORR (OR: 3.00, 95% CI: 2.05–4.38) (**Figure 4B**) and DCR (OR: 2.66, 95% CI: 1.70–4.16) (**Figure 4C**) at M6 were elevated by CSM-TACE treatment compared with cTACE treatment without significant heterogeneity among studies (all I^2^ = 0%, *P* > 0.05). Collectively, these findings exhibited that CSM-TACE treatment displayed superior CR, ORR and DCR at M1, M3, M6 over cTACE treatment.

### PFS and OS

**Figure 5:**
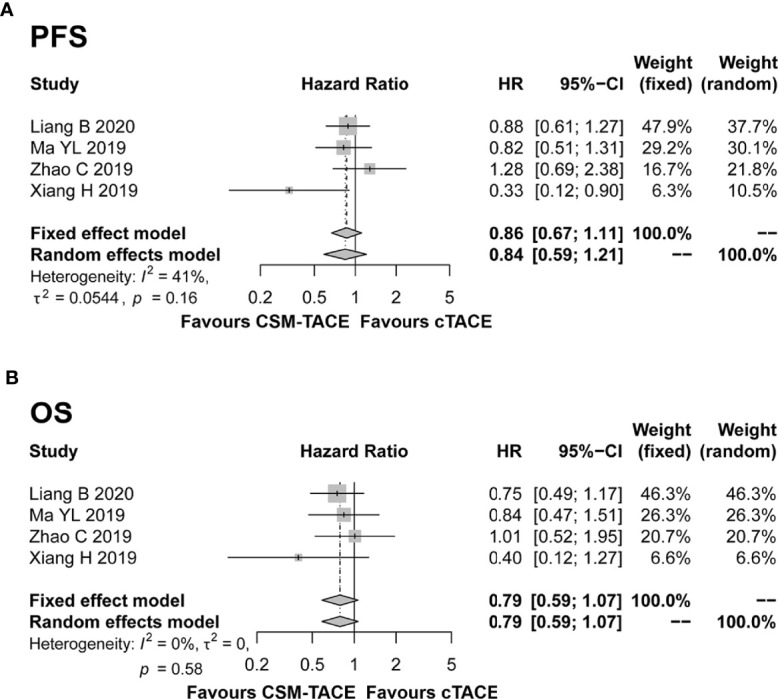
Forest plot comparing PFS **(A)** and OS **(B)** between CSM-TACE treatment and cTACE treatment. PFS, progression-free survival; OS, overall survival; CI, confidence interval; cTACE, conventional transarterial chemoembolization; CSM-TACE, CalliSpheres^®^ microspheres transarterial chemoembolization.

Four studies presented PFS and OS data, and were included in the pooled analysis. The pooled results showed a trend toward longer PFS (HR: 0.86, 95% CI: 0.67–1.11) (**Figure 5A**) and OS (HR: 0.79, 95% CI: 0.59–1.07) (**Figure 5B**) with CSM-TACE compared with cTACE, although these differences did not reach statistical significance. Meanwhile, there was no significant heterogeneity among studies (both I^2^ < 50%, both *P* > 0.05).

### Adverse Events

**Figure 6:**
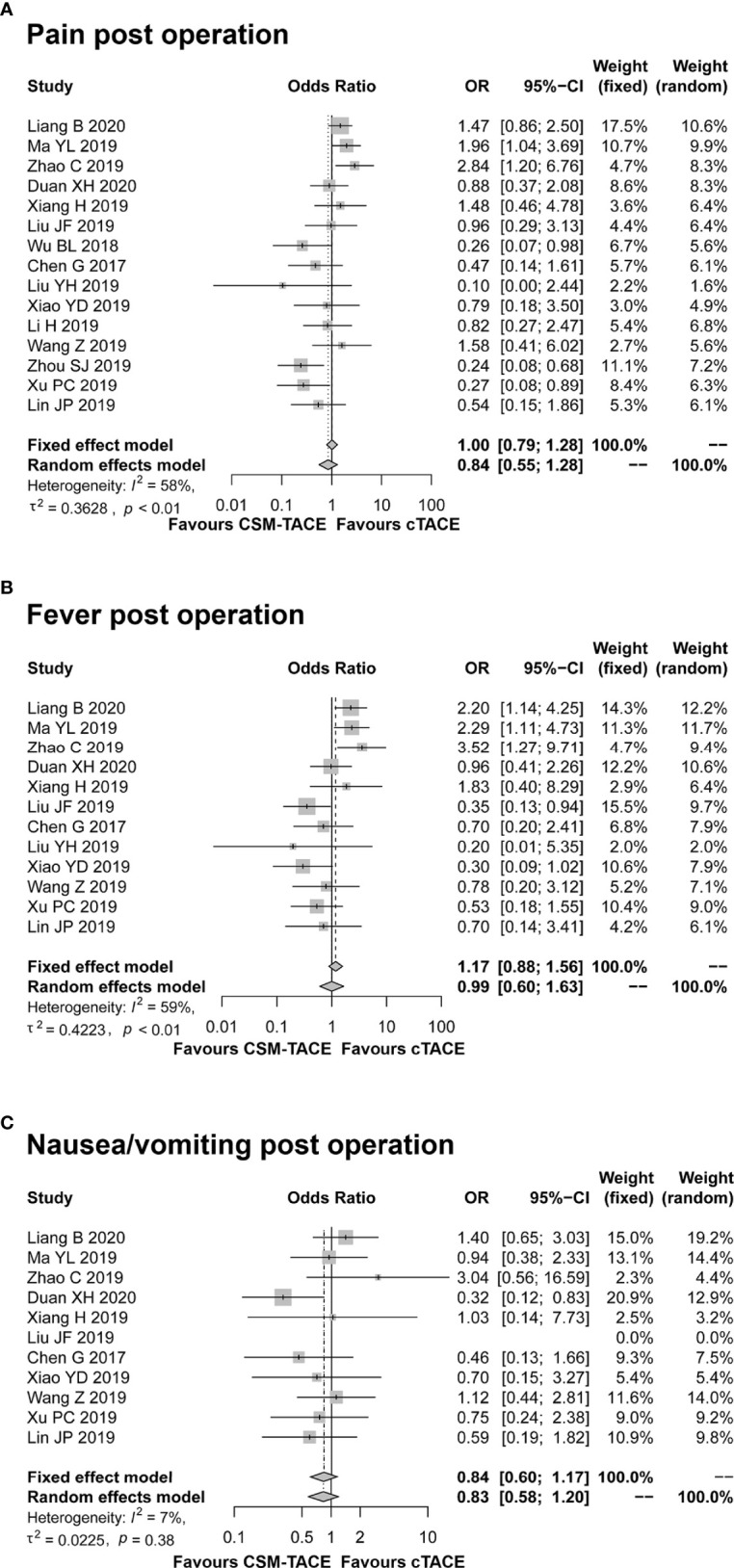
Forest plot comparing pain post operation **(A)**, fever post operation **(B)**, and nausea/vomiting post operation **(C)** between CSM-TACE treatment and cTACE treatment. CI, confidence interval; cTACE, conventional transarterial chemoembolization; CSM-TACE, CalliSpheres^®^ microspheres transarterial chemoembolization.

Pain, fever and nausea/vomiting after TACE treatment were reported in 15, 12, and 11 studies, respectively. The pooled analyses revealed that no difference was observed between CSM-TACE and cTACE treatment in post-treatment pain (OR: 0.84, 95% CI: 0.55–1.28) (**Figure 6A**), fever (OR: 0.99, 95% CI: 0.60–1.63) (**Figure 6B**) or nausea/vomiting (OR: 0.84, 95% CI: 0.60–1.17) (**Figure 6C**). No heterogeneity was found in the analysis of nausea/vomiting post treatment (I ^2^ = 7%, *P* = 0.38). In contrast, heterogeneity was identified in the analysis of post-treatment pain and fever (both I ^2^ > 50%, *P* < 0.01), and random effects model was adopted for their pooled analyses.

### Liver Function

**Figure 7:**
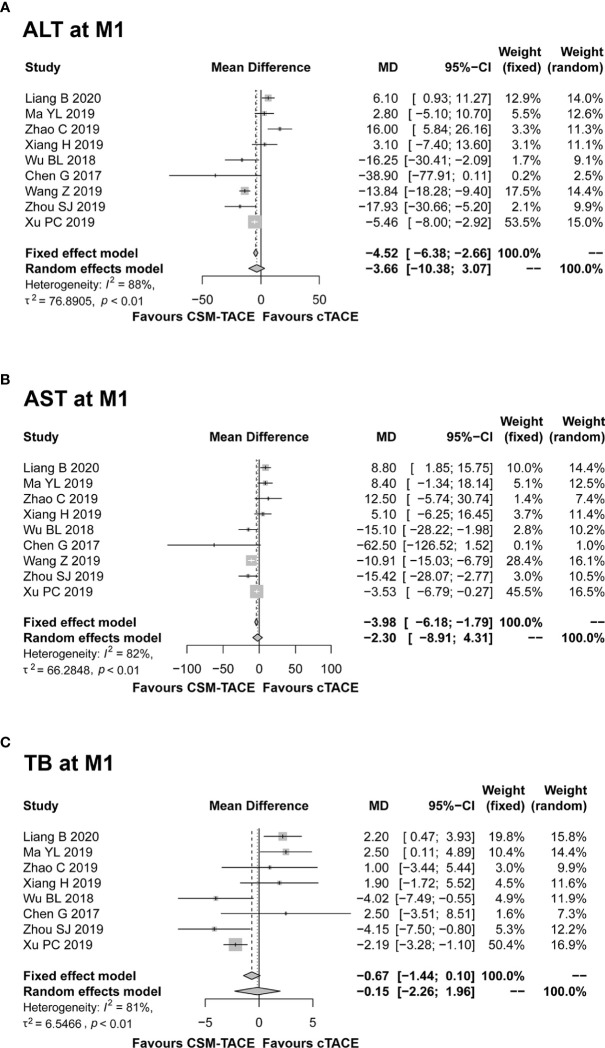
Forest plot comparing ALT at M1 **(A)**, AST at M1 **(B)**, and TB at M1 **(C)** between CSM-TACE treatment and cTACE treatment. ALT, alanine aminotransferase; M1, 1 month; AST, aspartate aminotransferase; TB, total bilirubin; CI, confidence interval; cTACE, conventional transarterial chemoembolization; CSM-TACE, CalliSpheres^®^ microspheres transarterial chemoembolization.

Nine studies presented data on ALT and AST at M1; 8 studies reported data on TB at M1. The pooled results showed that all ALT (MD: −3.66, 95% CI: −10.38–3.07) (**Figure 7A**), AST (MD: –2.30, 95% CI: −8.91–4.31) (**Figure 7B**) and TB (MD: −0.15, 95% CI: −2.26–1.96) (**Figure 7C**) at M1 were similar between two treatments. Of note, the heterogeneity among studies was identified (all I ^2^ > 50%, *P* < 0.01), and random effects model was used.

### Sensitivity Analysis

**Figure 8:**
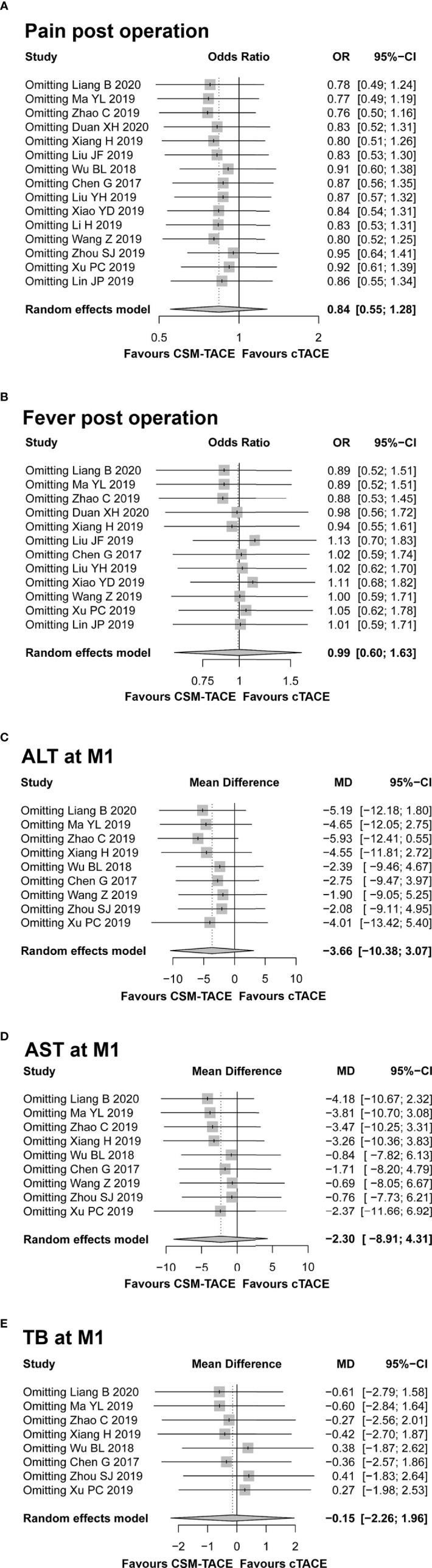
Sensitivity analysis for comparing pain post operation **(A)**, fever post operation **(B)**, ALT at M1 **(C)**, AST at M1 **(D)**, and TB at M1 **(E)** between CSM-TACE treatment and cTACE treatment. ALT, alanine aminotransferase; M1, 1 month; AST, aspartate aminotransferase; TB, total bilirubin; cTACE, conventional transarterial chemoembolization; CSM-TACE, CalliSpheres^®^ microspheres transarterial chemoembolization.

No significant heterogeneity was noted among studies in the analysis of CR, ORR, DCR at M1/M3/M6, PFS, OS, and nausea/vomiting post treatment (all I ^2^ < 50%, *P* > 0.05), whereas heterogeneity was found among studies in the analysis of post-treatment pain, fever, and 1-month ALT, AST, and TB (all I ^2^ > 50%, *P* < 0.01). Then, sensitivity analysis was conducted to assess the possible causes of heterogeneity, which showed that no single study could essentially change the pooled OR of post-treatment pain, fever and pooled MD of 1-month ALT, AST, and TB, demonstrating that the results of our meta-analysis were statistically stable (**Figures 8A–E**).

### Publication Bias

**Figure 9:**
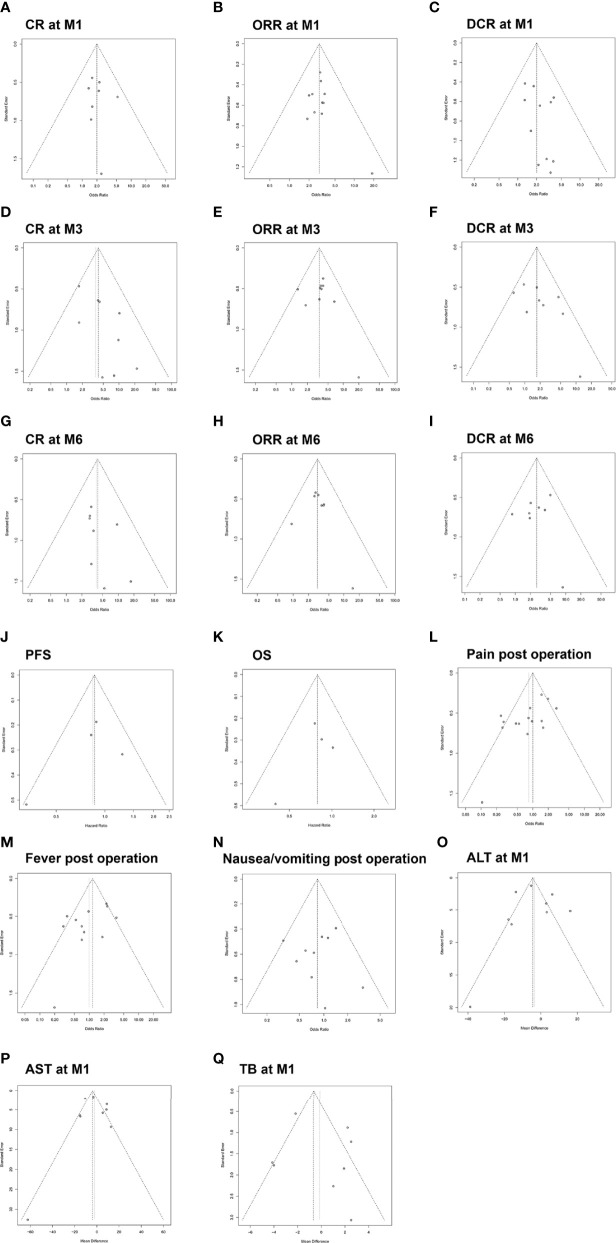
Funnel plot for the publication bias test of included studies with regard to CR at M1 **(A)**, ORR at M1 **(B)**, DCR at M1 **(C)**, CR at M3 **(D)**, ORR at M3 **(E)**, DCR at M3 **(F)**, CR at M6 **(G)**, ORR at M6 **(H)**, DCR at M6 **(I)**, PFS **(J)**, OS **(K)**, pain post operation **(L)**, fever post operation **(M)**, nausea/vomiting post operation **(N)**, ALT at M1 **(O)**, AST at M1 **(P)** and TB at M1 **(Q)**. CR, complete response; ORR, objective response rate; DCR, disease control rate; M1, 1 month; M3, 3 months; M6, 6 months; ALT, alanine aminotransferase; AST, aspartate aminotransferase; TB, total bilirubin.

The Begg’s funnel plots and Egger’s test displayed that there was no publication bias in the parameters under analysis except for pain post operation (**Figures 9A–Q**). The Begg’s and Egger’s test *p* values were summarized and listed in **Supplementary Table 1**.

## Discussion

Although drug eluting beads (DEB) have the ability to load chemotherapeutic agents and release them in a controlled mode, there is so far insufficient evidence to show that DEB-TACE is superior to cTACE (32–35). On comparison of CSM-TACE and cTACE for HCC, no meta-analysis has yet been reported. In the present meta-analysis, we initially searched formally published studies to comprehensively compare the efficacy and safety of CSM-TACE with that of cTACE for treating HCC patients. A total of 16 studies and 1,454 patients with HCC were included, of whom 722 were treated with CSM-TACE and 732 were treated with cTACE (14–16, 19–31). CR, ORR, DCR, PFS, OS, adverse events and liver function indexes were compared and analyzed. The pooled results of this study indicated that CSM-TACE was significantly superior to cTACE regarding CR, ORR, and DCR. Additionally, no difference of PFS, OS, adverse events or liver function was observed between two therapies.

Previous meta-analyses comparing treatment responses between DEB-TACE and cTACE for HCC have yielded conflicting results (32–35), which is likely caused by differences in included studies and population. Two previous meta-analyses included seven studies with 700 patients and nine studies with 877 patients, respectively, and concluded that DEB-TACE was associated with significantly improved tumor response rate and survival rate compared with cTACE (32, 33). In contrast, the other two previous meta-analysis included nine studies with 693 patients and 12 studies with 1,449 patients, respectively, and demonstrated that the two TACE procedures had equivalent results (34, 35). In the present study, we included all studies comparing the two TACE procedures and the meta-analysis of treatment responses showed that CSM-TACE increased 1-, 3-, and 6-month CR, ORR, and DCR compared with cTACE in HCC patients. The improved tumor responses are likely due to the superior pharmacokinetic properties and efficacy of CSM. CSM has the ability to load chemotherapeutic agents and release them in a controlled mode, allowing more sustained and tumor-selective delivery of chemotherapeutic drugs with lower systemic exposure. In addition, the calibrated CSM has shown permanent embolization (8). These advantages result in consistently improved tumor responses in the CSM-TACE group (8, 36). As for survival rates, the pooled results showed that CSM-TACE did not improve PFS and OS compared with cTACE in HCC patients. Notably, only four included studies provided PFS and OS data. Three of them demonstrated a non-significant trend in favor of the CSM-TACE group, which was likely to be explained by the fact that the relative short median follow-up durations (range: 9.9–11.4 months) might reduce the statistic power (16, 19, 21). In contrast, another study revealed that CSM-TACE improved PFS and OS compared with cTACE in HCC patients with statistical significance (20). As our statistical calculation was predominantly based on the pooled results for CSM-TACE versus cTACE, these three studies might contribute to an underestimation of the CSM-TACE survival benefit. Thereby, more studies with longer follow-up durations are needed in order to validate the survival benefit of CSM-TACE versus cTACE in HCC.

After treatment, post-operation abdominal pain, fatigue, fever and nausea/vomiting were the most commonly reported adverse events in both procedures, and the common adverse events can be ameliorated with symptomatic treatment (37). The pooled results of common adverse events displayed similar incidences of common adverse events, which was consistent with previous meta-analysis of DEB-TACE versus cTACE for HCC (18, 32, 33). One possible explanation for this could be that both TACE procedures employed the same transcatheter intra-arterial technique. It is well known that these common adverse events after TACE procedure are related to the chemotherapeutic agents-induced toxicity as well as the embolization-induced inflammatory response (postembolization syndromes). Although CSM-TACE and cTACE differed in the type and dose of chemotherapeutic drugs, the chemotherapy regimen administered in both TACE procedures was safe and well-tolerated by patients. In addition, both TACE procedures used similar embolization endpoints, and thus the two TACE procedures induced similar postembolization syndrome (8, 14, 20, 36). Furthermore, regarding post-operation liver function indexes, our meta-analysis disclosed no difference in ALT, AST or TB levels at 1 month after treatment between CSM-TACE and cTACE in HCC patients, which was likely explained by the similar minimal invasive techniques used in both TACE procedures (8, 14, 20, 36).

As heterogeneity was demonstrated among studies in the analysis of post-procedure pain, post-procedure fever, 1-month ALT, 1-month AST, and 1-month TB, we performed the sensitivity analysis to identify the possible causes of heterogeneity. Then we observed that the pool results were statistically stable in terms of pain, fever, ALT, AST, and TB. In addition, the risk of publication bias in this meta-analysis was assessed by the symmetry of funnel plots and Egger’s test. We found no evidence of publication bias among included studies regarding 1/3/6-month CR, 1/3/6-month ORR, 1/3/6-month DCR, PFS, OS, post-operation fever, post-operation nausea/vomiting, 1-month ALT, 1-month AST, and 1-month TB, but a certain publication bias among studies regarding post-operation pain in HCC patients. However, further meta-analyses including new related studies are still necessary for validation.

This meta-analysis offers comprehensive evidence showing the benefits of CSM-TACE over cTACE for treating HCC patients. However, the findings should be interpreted in the context of some limitations. First, the number of studies and subjects (ranging from 23 patients to 335 patients) included in this review were relatively small, and most of these studies were conducted in a single center, which might reduce the statistic power of the analysis. Second, only few randomized controlled trials regarding the efficacy and safety of CSM-TACE versus cTACE were available, and thus confounding factors might exist in the included studies and more randomized controlled trials were needed for further evidence. Third, a lack of uniform standards of chemotherapy agents and doses might lead to a bias of results. Lastly, differences in study designs, eligibility criteria for inclusion of HCC patients (such as tumor stage, Child-Pugh class, disease severity and treatment design) and experiences of interventional radiologists might lead to confounding bias.

## Conclusions

The results of this meta-analysis suggest that CSM-TACE displays superior treatment response, non-inferior survival profile and safety over cTACE in HCC patients, which might provide insights for supporting clinical decision-making and tumor management of HCC.

## Data Availability Statement

The original contributions presented in the study are included in the article/**Supplementary Material**. Further inquiries can be directed to the corresponding author.

## Author Contributions

BL: conception, design, data acquisition, analysis, interpretation and draft of the manuscript (review and editing). JM: methodology, data acquisition, investigation, software and formal analysis. SS, LZ and TS: data acquisition, analysis and interpretation. CZ: technical support and design of the research. All authors contributed to the article and approved the submitted version.

## Funding

This study was supported by the National Natural Science Foundation of China Grants (No. 81771950, 81471765).

## Conflict of Interest

The authors declare that the research was conducted in the absence of any commercial or financial relationships that could be construed as a potential conflict of interest.

## Abbreviations

ALT, alanine aminotransferase; AST, aspartate aminotransferase; CI, confidence interval; CNKI, China National Knowledge Infrastructure; CR, complete response; CSM, CalliSpheres^®^ microspheres; CSM-TACE, TACE with CalliSpheres^®^ microspheres; cTACE, conventional TACE; DCR, disease control rate; DEE-TACE, Drug-eluting embolic transarterial chemoembolization; HCC, hepatocellular carcinoma; HR, hazard ratio; MD, Mean difference; M1, 1 month; M3, 3 months; M6, 6 months; OR, odds ratio; ORR, objective response rate; OS, overall survival; PFS, progression-free survival; PRISMA, Preferred Reporting Items for Systematic Reviews and Meta-Analysis; TACE, transarterial chemoembolization; TB, total bilirubin; 95%CI, 95%confidence interval.
