# Supplementary material for: Treatment Response, Survival, and Safety of Transarterial Chemoembolization With CalliSpheres® Microspheres Versus Conventional Transarterial Chemoembolization in Hepatocellular Carcinoma: A Meta-Analysis
Source: Front Oncol. 2021 Mar 16;11:576232. doi: 10.3389/fonc.2021.576232 (PMC8008112; doi:10.3389/fonc.2021.576232)
Supplement: Supplementary file 1 [file Table_1.docx]

**Supplementary table 1**. Summary of *P* values by Begg’s test and Egger’s test

|  | Begg’s test (*P* value) | Egger’s test (*P* value) |
| --- | --- | --- |
| CR at M1 | 0.805 | 0.707 |
| ORR at M1 | 0.815 | 0.409 |
| DCR at M1 | 0.243 | 0.196 |
| CR at M3 | 0.297 | 0.054 |
| ORR at M3 | 0.788 | 0.513 |
| DCR at M3 | 0.144 | 0.101 |
| CR at M6 | 0.083 | 0.208 |
| ORR at M6 | 0.460 | 0.606 |
| DCR at M6 | 0.805 | 0.759 |
| PFS | 0.497 | 0.498 |
| OS | 1.000 | 0.499 |
| Fever post operation | 0.273 | 0.082 |
| Nausea/vomiting post operation | 0.788 | 0.961 |
| ALT at M1 | 0.404 | 0.861 |
| AST at M1 | 0.677 | 0.794 |
| TB at M1 | 0.805 | 0.515 |
| pain post operation | 0.151 | 0.014 |

CR, complete response; ORR, objective response rate; DCR, disease control rate; M1, 1 month; M3, 3 months; M6, 6 months; PFS, progression-free survival; OS, overall survival; ALT, alanine aminotransferase; AST, aspartate aminotransferase; TB, total bilirubin.
